# Mortality of malignant otitis externa: A prevalence meta-analysis

**DOI:** 10.5339/qmj.2025.53

**Published:** 2025-06-09

**Authors:** Adham A. Aljariri, Ahmad R. Al-Qudimat, Rani Hammoud, Abdulqadir J. Nashwan, Aisha Y. Larem, Mohamed B. Al Darwish, Hassanin Abdulkarim, Yasser Hamad, Yousra M. Khair, Amna S. Makawi, Hassan H. Ahmed, Ali Asaadi, Abdulsalam Alqahtani, Ahmad A. Abujaber

**Affiliations:** 1 Otolaryngology Department, Ambulatory Care Center (ACC), Hamad Medical Corporation (HMC), Doha, Qatar; 2 Department of Surgery, Surgical Research Section, Hamad Medical Corporation, Doha, Qatar; 3 Department of Quality and Patient Safety, Rehabilitation Institute, Hamad Medical Corporation, Doha, Qatar; 4 Department of Nursing, Hazm Mebaireek General Hospital, Hamad Medical Corporation, Doha, Qatar *Email: aalqudimat@hamad.qa

**Keywords:** Malignant, otitis, externa, mortality

## Abstract

**Introduction:**

Malignant otitis externa (MOE) is an aggressive infection of the external auditory canal and the underlying bony structures of the skull base. Predominantly caused by Pseudomonas, the treatment has shifted from surgical to medical, with antimicrobial therapy being primary, although surgical intervention may still be required. This review aims to provide global prevalence and mortality data on MOE to help institutions establish treatment benchmarks.

**Methods:**

A systematic review and meta-analysis followed the Preferred Reporting Items for Systematic Reviews (PRISMA) guidelines. Searches were completed in Scopus and PubMed Databases for articles on MOE mortality published between 1994 and 2022. Publications included data on MOE, mortality, and all genders.

**Results:**

A total of 22 studies involving 9,633 patients diagnosed with MOE were analyzed. The gender distribution was nearly equal, with 4,819 (50.1%) males and 4,814 (49.9%) females. The patients’ ages ranged from 18 to 90 years, with a mean age of 70.3 years. The pooled period prevalence of mortality due to MOE was estimated to be 18% (95% confidence interval: 6–30%), highlighting a significant mortality risk in patients with this condition. Heterogeneity across the studies was high (I^2^ = 99%, *p* < 0.001). Additionally, the prevalence of comorbidities was significant: 57.1% of patients were diabetic, 51% had hypertension, and other notable comorbidities included chronic pulmonary diseases (12.2%), liver disease (7.2%), and malignancies (3.4%). The most common microbiological cause was Pseudomonas aeruginosa (30%), followed by Staphylococcus aureus (10%). Surgical interventions were performed in 3.7% of cases, and cranial nerve involvement was reported in 9% of patients, primarily affecting the facial nerve (91%). Morbidity related to MOE was found to be 15.2%, and sepsis was a complication in 0.5% of cases. The results underscore the importance of addressing both comorbidities and mortality risks in managing MOE patients.

**Conclusion:**

This review highlights a significant global mortality rate of 18% in patients with MOE, with comorbidities like diabetes and hypertension contributing to worse outcomes. Despite current treatment advancements, mortality and morbidity remain substantial, stressing the need for early diagnosis, targeted interventions, and improved management strategies to enhance patient survival and outcomes.

## Introduction

Malignant otitis externa (MOE), also known as necrotizing otitis externa, is an erosive granulomatous infection affecting the soft tissue of the external ear canal, adjacent structures, and the skull base. Skull base osteomyelitis is a more accurate term, as this invasive infection erodes the temporal b one through Santorini’s fissures and the tympan mastoid fissure. Subsequent spread may occur to the jugular foramen and stylomastoid foramen. In advanced stages, it can involve cranial nerves, particularly the facial nerve. According to a study involving 23 patients, MOE was initially described by Toulmouche in 1838, with the term “malignant otitis externa” first coined by Chandler in 1968.^1–5^

The prevalence of MOE varies across age groups and is influenced by underlying pathogenic factors. Elderly individuals affected by this condition often have diabetes mellitus (DM), with reported rates of 90%–100% among MOE patients. Micro-angiopathy resulting from DM facilitates microbial invasion, leading to thrombophlebitis, thrombosis, and coagulative necrosis. The infection progresses from the external canal to involve adjacent bones and structures. In younger patients, MOE typically occurs in those who are immunocompromised due to conditions such as human immunodeficiency virus (HIV) infection, immunosuppressive therapy, or malignancy. MOE is exceptionally rare among pediatric patients, primarily affecting those with congenital immune deficiencies or malignancies.^4–6^ Pseudomonas aeruginosa is the predominant organism isolated in cases of MOE, identified in nearly 95% of instances according to many studies. Pseudomonas is not a normal inhabitant of the external ear canal but is believed to be associated with frequent ear irrigation for wax impaction therapy. Another predisposing factor for pseudomonal infection is an elevated pH in the external ear canal. Additionally, other organisms such as Staphylococcus aureus and various fungal species including Candida and Aspergillus have also been isolated as causative agents.^5–7^ Patients with MOE typically present with deep-seated otalgia, otorrhea, aural fullness, and often conductive hearing loss. In advanced stages, cranial nerve palsies and trismus may occur due to involvement of the temporomandibular joint. Diagnosis relies on a combination of clinical findings, elevated inflammatory markers, and radiological evidence of invasive infection, with or without bone erosion. Various radiological modalities have been employed to evaluate MOE patients; however, no single modality has been universally established as the gold standard or the optimal choice.^3,4,8^ Before the introduction of antibiotics as a cornerstone for MOE treatment, surgery was the treatment of choice for the disease, and it was associated with high morbidity. However, surgical treatment is still an option for cases refractory to antimicrobial therapy; the surgical treatment proposed ranges from debridement to mastoidectomy.^1–3^ Herein, this review is conducted to provide the prevalence mortality of this disease worldwide that specific institutions might utilize to establish treatment benchmarks.

Limited research has been conducted on the mortality and morbidity associated with MOE. In this meta-analysis, we aim to consolidate and synthesize the existing data to provide a comprehensive overview of the associated risks and outcomes. By aggregating the available evidence, we seek to offer a clearer understanding of the impact of MOE on patient health and inform future clinical practices and research directions.

## Methods

This review was done according to the Preferred Reporting Items for Systematic Reviews and Meta-analysis Guidelines (PRISMA).^4^ The protocol is registered in PROSPERO (International Prospective Register of Systematic Reviews) with a unique No. (CRD42022347272).

### Search strategy

A thorough search was completed in Scopus and PubMed Databases for articles on MOE mortality published between 1994 and 2022 using the terms MOE and mortality. Search applied for the Subject [MeSH] as follows: (“Malignant” [MeSH]) and “Otitis” [MeSH] and “Externa” [MeSH]) and “Mortality” [MeSH]. We were dedicated to prospective studies, retrospective studies, case reports, and series. All records were retrieved, including cohort studies, case reports, letters to the editor, and editorials. In-press, preprints, and accepted articles were also considered.

### Eligibility criteria

The patient, intervention, comparison, outcomes, study (PICOS) design summary of the study was as follows: (P): Adult patients, (I): patients diagnosed with malignant/necrotizing otitis externa, (C): Not applicable, (O): Mortality, (S): Observational studies (retrospective or prospective cohort studies). Articles were included in this work if they satisfy the subsequent conditions (a) Demographic information on patients with malignant/necrotizing otitis externa; (b) information on outcomes of patients with MOE such as mortality, or hospitalization. The following were excluded: (1) patients (less than 18 years); (2) non-malignant/necrotizing otitis externa patients; (3) Articles that have been published in English to date but have not been accepted, and were retrieved from scientific databases.

### Study selection

After careful screening and using the inclusion criteria, relevant articles were selected. Three authors (AAA, ARA, and MBA) evaluated and reviewed the texts of publications. Where there was a difference of opinions, the opinion of the senior author (OMA) was taken until an agreement was established.

### Data extraction

Many pertinent variables were extracted from selected articles such as the supplied data and based on the main stratification variables such as author, country, age range, data source, study timeframe, baseline population group, outcome (mortality), total sample, and others.

### Assessment of risk of bias

In this review, we used the Hoy criteria to assess the risk of bias in studies and offered a structured method to evaluate the validity of research findings based on various dimensions of study design and execution. This tool covers ten specific criteria, ranging from the representativeness of the target population and the sampling methods used, to the reliability and validity of the measurement instruments. Each criterion is scored as either ‘Yes’ (low risk of bias) or ‘No’ (high risk of bias), with the total score determining the overall risk of bias: low (0–3), moderate (4–6), or high (7–9).

### Statistical analysis

Meta-analysis was achieved for the prevalence of MOE mortality using Stata 17 software. Furthermore, a detailed synthesis was utilized to report the outcomes of the prevalence of MOE mortality. The q-value was used to find n heterogeneity between studies. A random-effect model was used based on the level of heterogeneity. Based on the Cochrane criteria, we used the random-effect model when the heterogeneity was over 50%. The event rate with a 95% confidence interval (CI) was calculated for each variable. Egger’s test and visual inspection of the funnel plot were used to assess publication bias.

## Results

### Study selection

**Figure 1:**
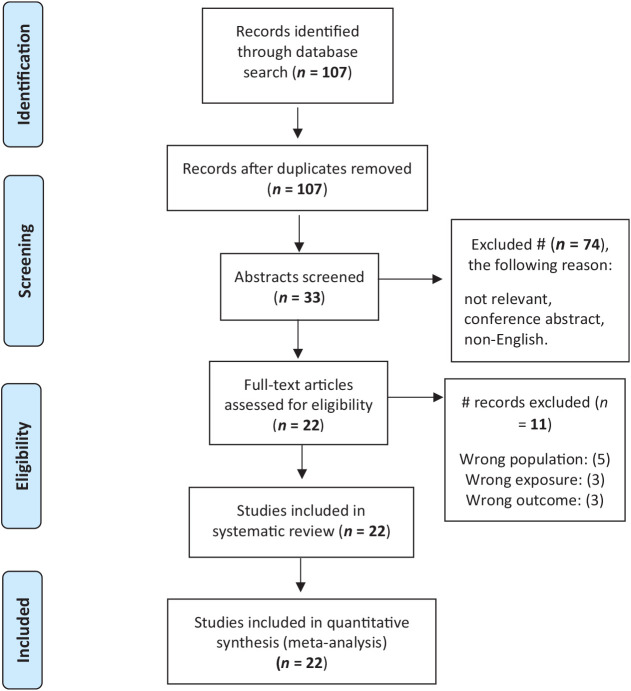
PRISMA diagram of literature search.

There were 107 studies found in the literature review. 74 were disqualified based on their titles. 11 were excluded due to inclusion criteria not being met (wrong population, wrong exposure, and wrong outcome). The remaining 22 were incorporated into our research study (Figure 1).

### Assessment of risk of bias

The selected studies were initially evaluated by the authors for external and internal validity, following the standards for bias assessment in prevalence and incidence studies.^5^ The bias assessment was performed for 22 articles included in the analysis. The studies reviewed consistently scored within the low risk-of-bias range, suggesting robust methodological quality and reliable findings (Table 1).

### Study characteristics

22 studies were included in this review with a total of 9,684 patients from both genders with a mean age of 70.3 years, who were diagnosed with MOE worldwide. The pooled prevalence was obtained from eight studies from the USA, Israel, Singapore, and Austria. The studies included the years from 1990 to 2021 (Table 2).

### Data analysis

Quantitative data were pooled using Stata software, and the results were expressed as pooled prevalence with corresponding 95% CIs. The robustness of the meta-analyses was evaluated using appropriate meta-analytical models based on the level of heterogeneity. Statistical heterogeneity was assessed using the I-squared (*I*^2^) statistic.

Given the significant heterogeneity commonly observed in prevalence and incidence meta-analyses, the random effects model was employed, as recommended by the Cochrane Methods Group.

Forest plots were utilized to present the incidence of chronic myelogenous leukemia along with the corresponding 95% CIs. Publication bias was assessed using a funnel plot, where the log event rate was plotted against the standard error.

### Pooled estimates

**Figure 2:**
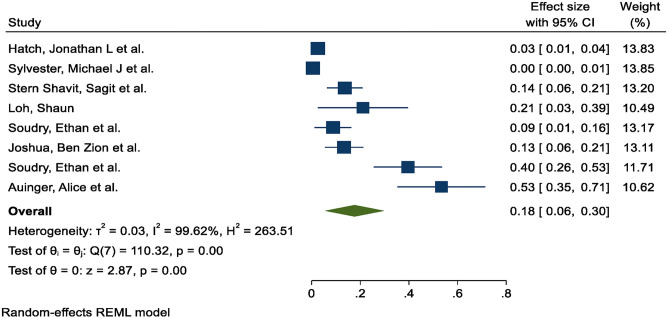
Estimation of the forest plots of prevalence for MOE.

Random effects period prevalence estimates for mortality due to malignant otitis externa (MOE) with 95% CI estimates as shown in Figure 2.

The random effects model was used to pool the estimated standardized incidence; it resulted in a pooled period prevalence of mortality due to malignant otitis externa 18% (95% CI: 6%–30%). The significant heterogeneity was met. The *I*^2^ value = 99% and H2 equals 263.51 with a *p*-value <0.001.

*Publication bias*

**Figure 3:**
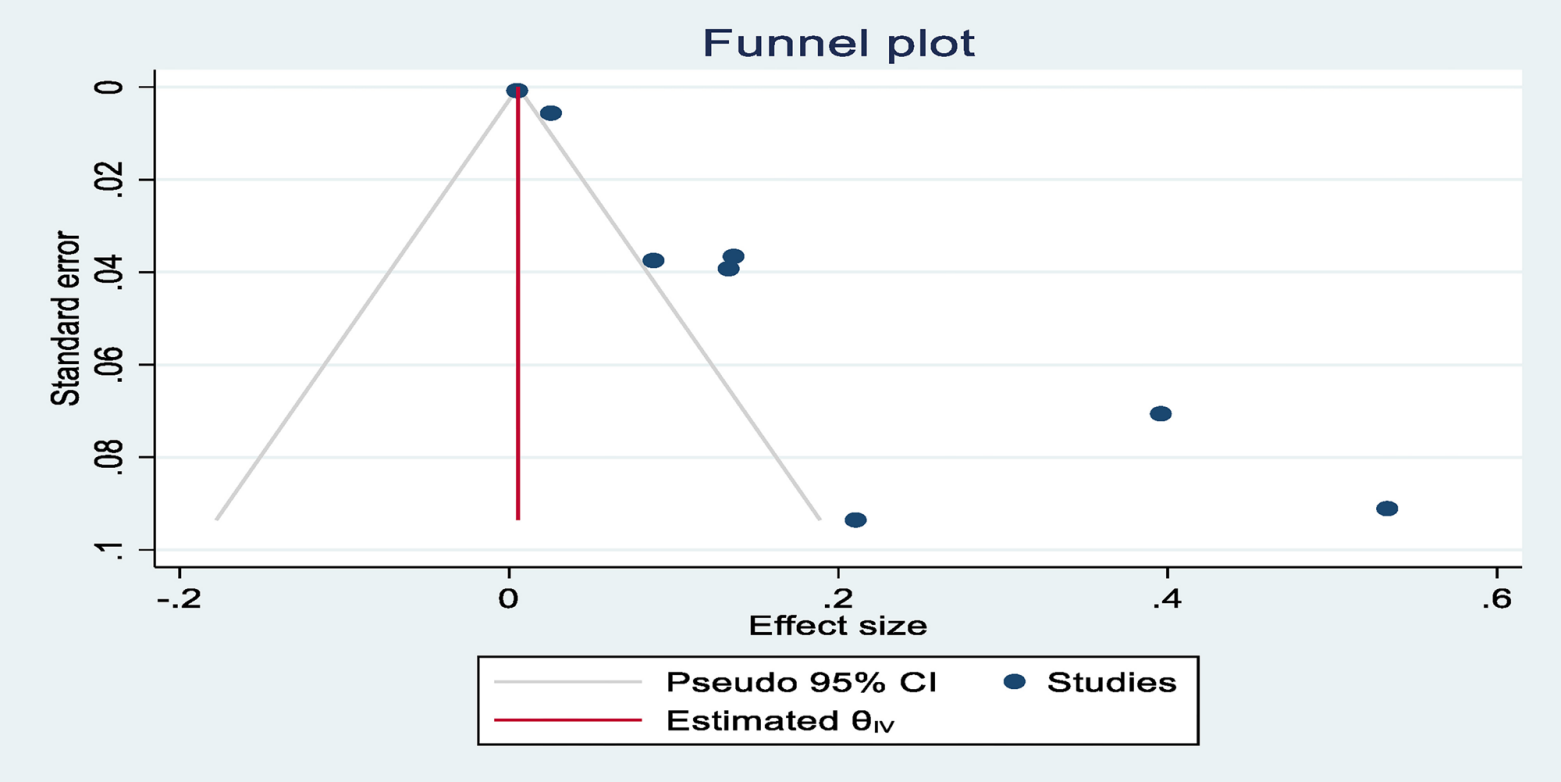
Publication bias.

Eight total studies (those that report CI) were included in this review. Asymmetry to the right was found in the plot, which proves the bias. Therefore, such bias can lead to misinterpretation of results and may ultimately lead to inaccurate conclusions (Figure 3).

### Patient demography and comorbidities

In our pool of data, a total of 9,545 have been included in our analysis, 4,758 (49.8%) males and 4,787 (50.2%) females; other studies reported male predominance with a 1.45:1 M:F ratio 5,436 (56.9%) of the subjects were diabetic, 4,877 (51%) have hypertension, other comorbidities as a liver disease were about 692 (7.2%), chronic pulmonary disease 1,167 (12.2), leukemia/lymphoma and other malignancies 332 (3.4%), rheumatoid arthritis 25 (0.2%), HIV/AIDS and immunodeficiency disorders 137 (1.4%), peripheral vascular disease 32 (0.3%), and history of CVA in 26 patients (0.2%). We found only in one report the statistically significant difference between mortality and weight loss for 628 patients (OR = 10.2; 95% CI 0.61–1.85; ES 1.23).^26^

### Microbiology

Causative organisms were not reported in some of the studies, in our results, Pseudomonas aeruginosa was found at 30%, Staphylococcus aureus followed by 10%, Aspergillus, Candida, and enterobacteria was also reported around 2% of the cases, other organisms as Streptococcus, group D Streptococcus, and Klebsiella were also reported.

### Interventions

348 (3.7%) of the patients underwent surgical procedures as a part of their treatment, procedures ranged from myringotomy, debridement, middle or inner ear biopsy, repair of the middle ear, external auditory canal reconstruction, radical mastoidectomy, cranial nerve decompression, mastoidectomy and excision of skull lesion. Central venous catheterization was a commonly performed procedure due to a prolonged antibiotic therapy regimen, and hyperbaric oxygen therapy was among the interventions used in patients with disease refractory to both medical and surgical treatments. Hyperbaric oxygen therapy is an accepted treatment modality. However, there are no specific recommendations regarding the duration of this therapy, and the available evidence on optimal treatment duration is limited and weak 41.

### Morbidity and mortality

In our results, morbidity and mortality were found to be 15.2% and 1.5%, respectively. Total morbidity associated with MOE was 15.2%, cranial nerve involvement was the most common complication associated with MOE, they were involved in 863 cases (9%), 91% of cranial nerve involvement was facial nerve VII, and the other 9% was IX, X, XI, and XII, sepsis was in 49 patients (0.5%), hospital readmission in 15 patients out of 9,545, other complications represented 5.5%.

## Discussion

MOE is considered a rare disease yet associated with high morbidity and mortality. Up to now, there are no exact reports about the incidence of this infection worldwide, however, some reports indicate an increase in incidence in some areas.

The morbidity and mortality of MOE may be related to the aggressiveness of the infection and its proximity to vital neurovascular structures, with the involvement of these structures often indicating a poorer prognosis, according to some studies.^1,26,27^ In this systematic review, the pooled mortality rate of 18% aligns with previous reports, which have estimated mortality rates ranging from 15% to as high as 50% in severe cases.^7,23,27^ Comparatively, Hatch et al.^7^ reported a mortality rate of 15%, emphasizing the variability in outcomes depending on the severity of cranial nerve involvement and comorbidities. These findings reaffirm the significant mortality burden associated with MOE, especially in patients with poorly controlled diabetes, which remains the most common risk factor.^7^

Complications of MOE are due to the direct spread of infection and involvement of the cranial nerves, the facial nerve (VII) being most affected, while other complications may develop from direct extension from the skull base to meninges and sigmoid sinus causing meningitis and sigmoid sinus thrombosis respectively, delay in diagnosis and treatment may lead to morbidity.^7,21,23,29^ Our study highlighted a high prevalence of cranial nerve involvement, particularly of the facial nerve, consistent with prior literature. Gonzalez et al. (2021) reported that cranial nerve involvement, particularly the facial nerve, increases mortality by up to 50%, demonstrating the need for early intervention.^27^ Hatch et al. also noted that facial nerve involvement and skull base osteomyelitis were associated with longer hospital stays and increased morbidity.^7^ In Chen et al.’s study, the facial nerve was the most frequently affected cranial nerve (38.46%), and multiple cranial nerve involvement was significantly correlated with higher mortality rates.^23^ In our review, cranial nerve involvement was observed in 9% of cases, primarily affecting the facial nerve (91%), corroborating earlier findings that this complication is a critical determinant of poor prognosis.^7,27^ Another important aspect is the microbiological profile of MOE. Our review found Pseudomonas aeruginosa as the most common causative pathogen in 30% of cases. This is consistent with Treviño González et al. (2021), who reported Pseudomonas as the predominant pathogen, affecting up to 90% of MOE cases.^27^ However, the emergence of other pathogens such as methicillin-resistant Staphylococcus aureus and fungi, as noted by Long et al. (2020), suggests a shift in the microbial landscape, which may complicate treatment strategies.^7^ This highlights the importance of obtaining accurate microbiological cultures to guide appropriate antimicrobial therapy.

The management of MOE remains challenging, especially in patients with underlying comorbidities. As noted in our study, 57.1% of patients were diabetic. Furthermore, two reposts documented that the estimates up to 90% of MOE patients have diabetes.^7,27^ Also, our study found that diabetes was the most prevalent comorbidity, affecting 56.9% of MOE patients. This aligns with previous reports, where DM has been consistently identified as the most significant risk factor for MOE.^7,27^ Chen et al. similarly reported that all patients in their study had diabetes, although the study found no significant correlation between the duration of diabetes or the level of glucose tolerance and survival outcomes.^23^ The pathophysiology of MOE in diabetic patients involves microangiopathy and an impaired immune response, which predispose these individuals to severe infections and increased mortality.^27^ In addition, hypertension (51%) and chronic pulmonary disease (12.2%) were also common among MOE patients, further highlighting the impact of systemic health on disease progression and outcomes.

Finally, the findings of this review underscore the significant mortality and morbidity associated with MOE, particularly in diabetic and immunocompromised populations. The high prevalence of cranial nerve involvement and the evolving microbial profile further complicate management, necessitating a multidisciplinary approach. Early diagnosis, aggressive treatment, and strict control of comorbid conditions are essential for improving patient outcomes. Future studies should focus on optimizing treatment protocols and exploring novel therapies, such as hyperbaric oxygen therapy, which has shown promise in refractory cases but lacks robust clinical trial data.^27^

### Limitations

In our review, several potential limitations deserve mention. First, our data was collected from multiple studies with smaller to medium sample sizes that had a relatively low risk of bias. However, most patients came from two large retrospective studies in the USA, with 8,300 and 786 patients. Other studies have fewer than 100 patients, so in the final calculations, the results affected most of these two studies. Second, MOE diagnosis is uncommon; therefore, it is unexpected to find more comprehensive prospective studies. Third: Many different institutions published the studies over several decades; variations in technology, diagnostic, and mortality confirmation criteria may serve as a confounder.

This review included a total of eight studies (those reporting CIs). The plot showed a rightward asymmetry, indicating a potential bias. Such bias can result in misinterpretation of the results and may ultimately lead to inaccurate conclusions.

## Conclusion

The global prevalence of MOE is accompanied by a significant mortality rate, with an estimated pooled mortality prevalence of 18%. The findings indicate that comorbidities, particularly diabetes and hypertension, play a crucial role in the clinical outcomes of MOE patients. High levels of heterogeneity among the studies suggest variability in regional data and treatment approaches. Despite advancements in management, the morbidity and mortality associated with MOE remain substantial, emphasizing the need for early diagnosis, effective treatment strategies, and comprehensive management of comorbid conditions to improve patient outcomes. Further research is warranted to refine treatment protocols and reduce the mortality risk in this population.

## Conflicts of interest

The authors report no conflicts of interest.

## Authors’ contribution

AAA, ARA, AYL, MBA, YMK, and ASM performed the literature search, and collected and interpreted the data. AAA, ARA, AA, OMA, and RMA drafted the work and contributed to the writing of this manuscript. ARA and YH performed the meta-analysis. AAA, ARA, HHA, AYL, and RMA edited and drafted the final version of this manuscript. AAA, ARA, and RMA reviewed the final version to be published.

## Ethical consideration

No ethical approval was required for this review.

## Figures and Tables

**Table 1.** Assessment of bias per Hoy criteria described.

**Author, years**	**1**	**2**	**3**	**4**	**5**	**6**	**7**	**8**	**9**	**10**	**Score**	**Risk of bias**
Pichon et al.^6^	Y	Y	N	Y	Y	Y	Y	Y	Y	Y	1	Low
Hatch et al.^7^	Y	Y	Y	Y	Y	Y	Y	Y	Y	Y	0	Low
Sylvester et al.^8^	Y	Y	Y	Y	Y	Y	Y	N	N	Y	2	Low
Stern et al.^9^	Y	Y	Y	Y	Y	Y	Y	Y	Y	N	1	Low
Baig et al.^10^	Y	Y	N	Y	Y	Y	Y	Y	Y	N	2	Low
Loh and Loh^11^	Y	Y	Y	Y	Y	Y	Y	Y	Y	N	1	Low
Soudry et al.^12^	Y	Y	Y	Y	Y	Y	Y	Y	Y	N	1	Low
Joshua et al.^13^	Y	Y	Y	Y	Y	Y	Y	Y	Y	N	1	Low
Soudry et al.^14^	Y	Y	Y	Y	Y	Y	Y	Y	Y	Y	0	Low
Mani et al.^15^	Y	Y	Y	Y	Y	Y	Y	Y	Y	N	1	Low
Agada et al.^16^	Y	Y	Y	Y	Y	Y	Y	Y	Y	N	2	Low
Singh et al.^17^	Y	Y	N	Y	Y	Y	Y	Y	Y	N	2	Low
Lancaster et al.^18^	Y	Y	N	Y	Y	Y	Y	Y	Y	N	2	Low
Harley et al.^19^	Y	Y	N	Y	Y	Y	Y	Y	Y	N	2	Low
Krishnamoorthy et al.^20^	Y	Y	N	Y	Y	Y	Y	Y	Y	N	2	Low
Ridder et al.^25^	Y	Y	Y	Y	Y	Y	Y	Y	Y	N	1	Low
Sekar et al.^26^	Y	Y	N	Y	Y	Y	Y	Y	Y	Y	1	Low
Bhasker et al.^21^	Y	Y	Y	Y	Y	Y	Y	Y	Y	Y	0	Low
Auinger et al.^22^	Y	Y	Y	Y	Y	Y	Y	N	N	Y	2	Low
Stern et al.^9^	Y	Y	Y	Y	Y	Y	Y	Y	Y	N	1	Low
Chen et al.^23^	Y	Y	Y	Y	Y	Y	Y	Y	Y	N	1	Low
Glikson et al.^24^	Y	Y	Y	Y	Y	Y	Y	Y	Y	N	1	Low
**Risk-of-bias items** 1. Was the study’s target population a close representation of the national population in relation to relevant variables, e.g., age, sex, and occupation? 2. Was the sampling frame a true or close representation of the target population? 3. Was some form of random selection used to select the sample, or was a census undertaken? 4. Was the likelihood of non-response bias minimal? 5. Were data collected directly from the subjects (as opposed to a proxy)? 6. Was an acceptable case definition used in the study? 7. Was the study instrument that measured the parameter of interest shown to have reliability and validity? 8. Was the same mode of data collection used for all subjects? 9. Were the numerator(s) and denominator(s) for the parameter of interest appropriate Summary of the overall risk of study bias							**Risk-of-bias levels** Yes = Low Risk= 0 No = High Risk= 1			**Risk-of-bias results** Low Risk = 0–3 Moderate Risk = 4–6 High Risk = 7–9		

**Table 2.** Summary of relevant research studies.

**Author, year**	**Country**	**Study design**	**No. of Patients**	**Age**	**Enrollment period**
Pichon et al.^6^	France	Case report	1	72	(2019)
Hatch et al.^7^	USA	Retrospective	786	(<18)–(>84)	(2012–2015)
Sylvester et al.^8^	USA	Retrospective	8,300	54.1 ± 20.4	(2002–2013)
Stern et al.^9^	Israel	Retrospective	88	73 ± 11.5	(1990–2013)
Baig et al.^10^	Pakistan	Case report	1	37	(2013)
Loh and Loh^11^	Singapore	Retrospective	19	69.1	(2006–2011)
Soudry et al.^12^	Israel	Retrospective	57	76	(1990–2008)
Joshua et al.^13^	Israel	Retrospective	75	(71.8 ± 19) (54.4 ± 11)	(1990–2003)
Soudry et al.^14^	Israel	Retrospective	48	78	(1990–2004)
Mani et al.^15^	UK	Retrospective	23	71 (39–87)	10 years
Agada et al.^16^	UK	Case report	1	70	(2006)
Singh et al.^17^	Oman	Case series	3	(54–74)	(2005)
Lancaster et al.^18^	UK	Case report	1	58	(1999)
Harley et al.^19^	USA	Case report	1	20	(1994)
Krishnamoorthy et al.^20^	Malaysia	Case report	1	60	(2019)
Ridder et al.^25^	Berlin	Retrospective	20	72	(2004–2011)
Sekar et al.^26^	India	Retrospective	79	66.30 ± 10.29	(2015–2021)
Bhasker et al.^21^	UK	Retrospective	11	38–97	(2004–2012)
Auinger et al.^22^	Austria	Retrospective	30	73.1 ± 13.5	(1993–2015)
Stern et al.^9^	Israel	Retrospective	88	73 ± 11.5	(1990–2013)
Chen et al.^23^	Taiwan	Retrospective	26	63.7 ± 10.2	(1993–2005)
Glikson et al.^24^	Israel	Retrospective	25	Ns	(2009–2015)
**Total**			**9,684**	
